# mirna-383-5p Functions as an Anti-oncogene in Glioma through the Akt/mTOR Signaling Pathway by Targeting VEGFA

**DOI:** 10.2174/1568009623666230817102104

**Published:** 2024-02-13

**Authors:** Yan Liu, Zhen Wang, Zhi Tang, Yao Fu, Lei Wang

**Affiliations:** 1 Department of Neurosurgery, Hunan Cancer Hospital and the Affiliated Cancer Hospital of Xiangya School of Medicine, Central South University, No. 283 Tongzipo Road, Yuelu District, Changsha, 410006, Hunan, China;; 2 Department of Neurology, Changsha Central Hospital, University of South China, No.161 Shaoshan Road, Yuhua District, Changsha, 410007, Hunan, China;; 3 Department of Neurosurgery, Yiyang Central Hospital, No.118 North Kangfu Road, Heshan District, Yiyang, 413000, Hunan, China;; 4 Changsha Medical University, No.1501 Leifeng Road, Wangcheng District, Changsha, 410219, Hunan, China

**Keywords:** Glioma, miRNA383-5p, VEGFA, Akt/mTOR pathway, cytometry, cell apoptosis

## Abstract

**Background:**

Previously, we have screened 59 differentially expressed miRNAs and 419 mRNAs in the glioblastoma samples that have been compared to the peritumoral tissues using bioinformatics analyses, which included miRNA-383-5p and vascular endothelial growth factor A (VEGFA). miRNA-383-5p and VEGFA/Akt/mTOR pathway play important regulatory roles in the malignant biological behavior of glioma.

**Methods:**

Glioma cell lines, U87 and U251, were collected for *in vitro* experiments. miRNA-383-5p and VEGFA expression levels were detected with qRT-PCR and WB. The protein expressions of Akt, mTOR, and VEGFR in U87 and U251 were detected with WB. The effect of miRNA-383-5p on the VEGFA activity was verified by dual-luciferase reporter assay. CCK-8 was used to examine the U87 and U251 cells’ inhibition. Flow cytometry and transwell assays were used to detect cell apoptosis and invasion, respectively.

**Results:**

Our research data indicated overexpression of miRNA-383-5p to suppress malignant biological behavior, which was manifested as promoting the apoptosis of U87 and U251 cells and inhibiting invasion, proliferation, and metastasis. VEGFA is one of the downstream target genes of miRNA-383-5p. miRNA-383-5p could inhibit the expression of VEGFA and Akt/mTOR signaling pathways. Overexpression of VEGFA can reverse the inhibitory effect of miRNA-383-5p and reactivate the Akt/mTOR signaling pathway.

**Conclusion:**

Our results indicate that miRNA-383-5p functions as an anti-oncogene by inhibiting the VEGFA/Akt/mTOR signaling pathway in glioma cells. These data provide potential therapeutic targets for glioblastoma.

## 1 INTRODUCTION

Approximately 100,000 people worldwide are diagnosed with diffuse glioma each year [1]. High-grade glioblastoma (GBM) accounts for 57% of all gliomas. Despite substantial advances in treatment, patients with this disease continue to have a poor prognosis and a median survival of 2 years or less. Tan *et al.* suggested that future research should focus on deepening our understanding of the underlying molecular biology of glioblastoma before searching for potential drug targets [2]. Therefore, more effective diagnostic and therapeutic methods are urgently needed.

In previous works, we have screened 59 differentially expressed miRNAs and 419 mRNAs by using bioinformatics tools. We have found some miRNAs to act as anti-oncogenes in glioma patients [3]. In a subsequent study, we have validated miRNA-139-5p, miRNA-338-3p, and miRNA-138-5p to be anti-tumor factors [3-5]. There are some other unproven miRNAs that may play an important role in the regulation of malignant biological behavior in gliomas, such as miRNA-383-5p.

MicroRNAs (miRNAs) are considered promising tumor markers and therapeutic targets due to their ability to silence the expression of target mRNAs through post-transcriptional regulation [6, 7]. Over the past decade, a series of studies have shown that miRNAs may play a potential role in immunotherapies against cancer [8, 9]. For example, a significant therapeutic effect has been proven in the treatment of human cancers using PD-L1 inhibitors, which target the immune checkpoint, and miR-34a is a natural inhibitor of PD-L1 [10, 11]. A meta-analysis study showed that miRNAs can serve as potential diagnostic markers for glioma [12]. Detection of specific miRNAs in cerebrospinal fluid and brain tissue was found to have a high accuracy in the diagnosis of glioma. Furthermore, aberrant expression of miRNAs was observed in multiple steps of glioma development, including proliferation, recurrence, and metastasis [13, 14].

In a previous study, we found that miRNA-383-5p may be an important factor in regulating the malignant biological behavior of glioma using a bioinformatics tool, and its downstream target was predicted to be vascular endothelial growth factor A (VEGFA). Therefore, we hypothesized that low levels of miRNA-383-5p could promote the malignant biological behavior of glioma by regulating the expression of VEGFA, and overexpression of VEGFA could reverse the effect, thus potentially identifying a therapeutic target.

## 2 MATERIALS AND METHODS

### 2.1 Cell Lines

Human glioblastoma multiforme cell lines, U87 and U251, were purchased from BeNa Culture Collection (BNCC, China). Both U87 and U251 cells were cultured in a medium containing 10% fetal serum (FBS, Gibco, USA). Cells were cultured at 37°C in an incubator containing 5% CO_2_.

### 2.2 Cell Transfection

A negative control (NC), a miRNA-383-5p mimic, and a pcDNA3.1-VEGFA plasmid overexpressing VEGFA were purchased from Changsha Honorgene Co., Ltd. (Hunan, China). Lipofectamine 2000 reagent (Invitrogen, Carlsbad, CA, USA) was used to transfect U87 and U251 cells according to the manufacturer's protocol.

### 2.3 qRT-PCR

Trizol total RNA extraction reagent (Thermo, USA) was used to extract the total RNA from U87 or U251 cells after transfection, according to the manufacturer's instructions. Total RNA (200 ng) was reverse transcribed by qRT-PCR using a SuperRT RT kit (CWBio, China) and SYBR PCR Master Mix (CWBio, China). PCR was set at 40 cycles of 95°C for 10 min, 95°C for 15 s, and 60°C for 30 s for initial denaturation. All experiments were repeated three times. Expression fold changes were calculated using the 2-ΔΔCt method. All primer sequences are listed in Table **1**.

### 2.4 Western Blot Analysis

Post-transfection, U87 or U251 cells were lysed with buffer containing RIPA for 30 min on ice and then centrifuged at 12,000 g/min for 20 min at 4°C. Cell lysates were assessed for total protein concentration using the BCA protein assay kit. Subsequently, 20 μg protein samples were separated on 10% SDS-PAGE using the Bio-Rad Bis-Tris Gel System (Bio-Rad, CA, USA) and transferred to polyvinylidene fluoride membranes (Millipore, Danvers, MA, USA). Membranes were blocked with 5% non-fat milk for 60 min at room temperature. The primary antibodies used were VEGFA (1:500, Proteintech, USA), VEGF2 (1:500, Proteintech, USA), Akt (1:5000, Proteintech, USA), mTOR(1:5000, Proteintech, USA), and β-actin (1:5000, Proteintech, USA); the corresponding secondary antibodies were anti-mouse and anti-rabbit (Proteintech, USA). Primary antibodies against VEGFA were purchased from Bioss Co., Ltd. (Beijing, China), and primary antibodies against VEGFA, Akt, mTOR, and β-actin were purchased from Proteintech, USA. Finally, proteins were detected by enhanced chemiluminescence (ECL).

### 2.5 Cell Viability Assay

Cell proliferation was detected using the Cell Counting Kit-8 (CCK-8; Tongren, Japan). U87 or U251 cells were seeded on 96-well plates 6 hours after transfection and were cultured with 100 μL of cell culture medium. After 48 h of culture, 10 μL of CCK-8 was added to the cell culture medium and incubated at 37°C for 4 h. Absorbance at 450 nm was measured using a plate reader (BioTek; Huisong, Shenzhen, China).

### 2.6 Colony Formation Assay

U87 or U251 cells were seeded in 6-well plates (Nunc, Roskilde, Denmark) at a density of 400 cells per well 6 hours after transfection. After 14 days of culture, cell colonies were fixed with alcohol, treated with 0.3% crystal violet solution (Sangon, Songjiang, Shanghai, China) for 0.5 h, and washed twice with deionized water.

### 2.7 Apoptosis Assay

The apoptosis rate of cells from different treatment groups was examined using the Apoptosis Detection Kit (KGA108, KeyGen BioTECH, China). Cells were washed 3 times with PBS (Hyclone, USA) and centrifuged. The harvested cells were suspended in 500 μL of binding buffer and centrifuged at 2000 rpm for 5 minutes before the addition of 500 μl of binding buffer, 5 μl of Annexin V-APC, and 5 μl of propidium iodide.

### 2.8 Transwell Assay

Transwell assays were used to assess migration and invasion of U87 or U251 cells 6 hours after transfection. Transwell chambers (Corning, Shanghai, China) were used to measure migration. Matrigel (BD, USA) was thawed at 4°C overnight, and then 100 µL of diluted Matrigel was added to the chamber. After that, 200 μL of sterile medium was added to the upper chamber, and at the same time, 500 μL of 10% FBS DMEM was added to the lower chamber. A total of 2 × 10^5^ collected cells were added to the upper chamber and cultured in the incubator for 48 h. Invasion chambers were subsequently removed and cells on polycarbonate membranes were fixed with 4% paraformaldehyde, and then stained with 0.1% crystal violet in acetic acid after soaking and de-staining. Cells were measured with a microplate analyzer in three random fields at an absorbance (OD) of 550 nm. Experiments were repeated three times.

### 2.9 Dual-luciferase Reporter Gene Assay

The Luciferase Assay System Kit (Promega, Madison, WI, USA) was used to detect luciferase activity in U87 and U251 cells during log growth. Dual-luciferase expression plasmids for the 3’UTR of the human VEGFA wild-type gene or the 3’UTR of the VEGFA mutant sequence (VEGFA WT or VEGFA MUT) were purchased from Honorgene (Aono Genomics, Longshah Biotechnology Ltd). Cells were seeded in 24-well plates and co-simulated with NC simulator or miRNA-383-5p simulator using Lipofectamine 2000 reagent (Invitrogen Co., Carlsbad, CA, USA) and VEGFA expression vector. Lipofectamine 2000 reagent (Invitrogen, Carlsbad, CA, USA) and VEGFA expression vector were co-transfected into NC mimics or miRNA-383-5p mimics. After 48 hours of incubation according to the manufacturer's instructions, the transfected cells were harvested and a luciferase activity assay was conducted according to the protocol of the Dual-Glo Luciferase Assay System Kit.

### 2.10 Statistical Analysis

All data have been presented as mean ± standard deviation of three independent experiments. Student’s t-test was performed to determine differences between the two groups. Analysis of variance was used for multiple group comparisons. *P* < 0.05 was considered to be statistically significant. Statistical and graphical analysis was performed using GraphPad Prism 5.

## 3 RESULTS

**Fig. (1):**
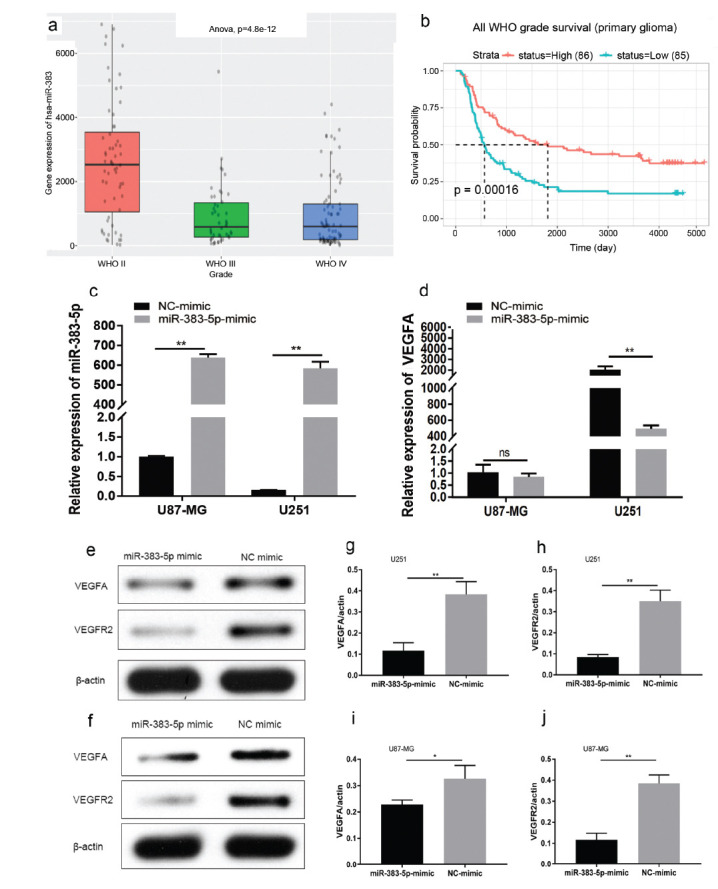
(**a**) The expression of miRNA-383-5p was found to be negatively correlated with the grade of glioma. (**b**) The prognostic value of miRNA-383-5p in gliomas of various grades. (**c**) miRNA-383-5p transfection increased miRNA-383-5p levels in U87 and U251 cells compared to NC-transfected cells. (**d**) qRT-PCR analysis of VEGFA expression in U251 and U87 cells treated with miRNA-383-5p mimic. (**e-h**) Western blotting was used to detect protein levels of VEGFA and VEGFR2 in U251 cells after transfection. (**f-j**) Western blotting was used to detect protein levels of VEGFA and VEGFR2 in U251 cells after transfection. Each experiment was repeated at least three times. ^∗^*P* < 0.05, ^∗∗^*P* < 0.01.

The expression of miRNA-383-5p has been found to be decreased in glioma and VEGFA may be a downstream target. Since our previous data found miRNA-383-5p to be abnormally expressed in gliomas, we searched the Chinese Glioma Genome Atlas (CGGA) database and found miRNA-383-5p to be negatively correlated with WHO grade of glioma; the higher the glioma grade, the lower the expression of miRNA-383-5p [3, 14] (Fig. **1a**). In addition, higher miRNA-383-5p expression has been found to be associated with higher patient survival (Fig. **1b**). To further explore the potential role of miRNA-383-5p in glioma cells, U251 and U87 cells were infected with lentiviruses expressing miRNA-383-5p or miRNA-NC. We performed qRT-PCR to evaluate the transfection. Compared to the control and LV-miRNA-383-5p cells, we found the expression of miRNA-383-5p to be significantly increased in U251 and U87 glioma cells in the LV-miRNA-383-5p group (Fig. **1c**). Transfection of the miRNA-383-5p mimic significantly increased VEGFA mRNA expression in U251 cells, but not in U87 cells (Fig. **1d**). Protein levels of VEGFA and its receptor VEGFR2 were detected using western blotting. The expressions of VEGFA and VEGFR2 have been found to be significantly decreased after transfection with miRNA-383-5p mimic (Figs. **1e - j**).

### 3.1 miRNA-383-5p Inhibits Glioma Cell Proliferation and Invasion While Promoting Apoptosis

**Fig. (2):**
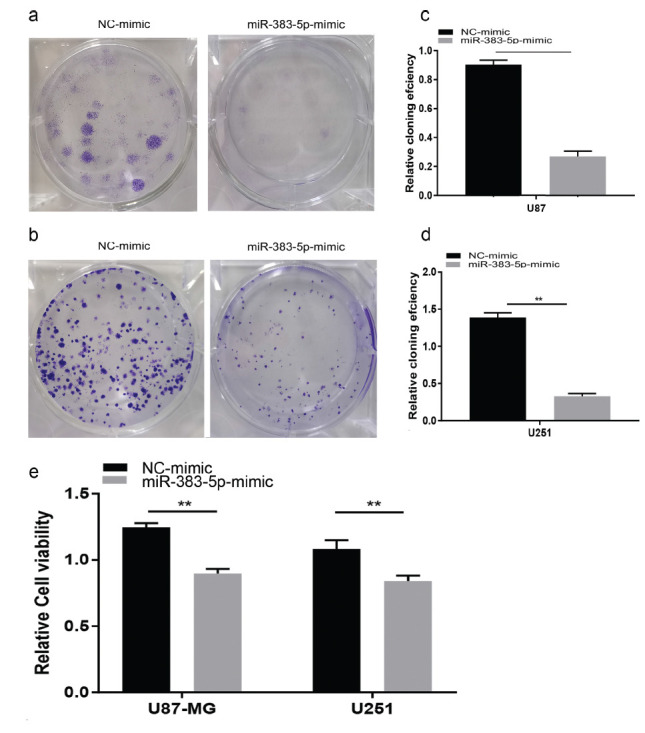
**(a-d)** Colony formation assays suggest that miR-383-5p mimic results in decreased U251 cell proliferation. (**e**) CCK8 experiments suggest that miR-383-5p mimic transfection reduces the cell viability of U87 and U251 cells. Each experiment was repeated at least three times. ^∗^*P* < 0.05, ^∗∗^*P* < 0.01.

**Fig. (3):**
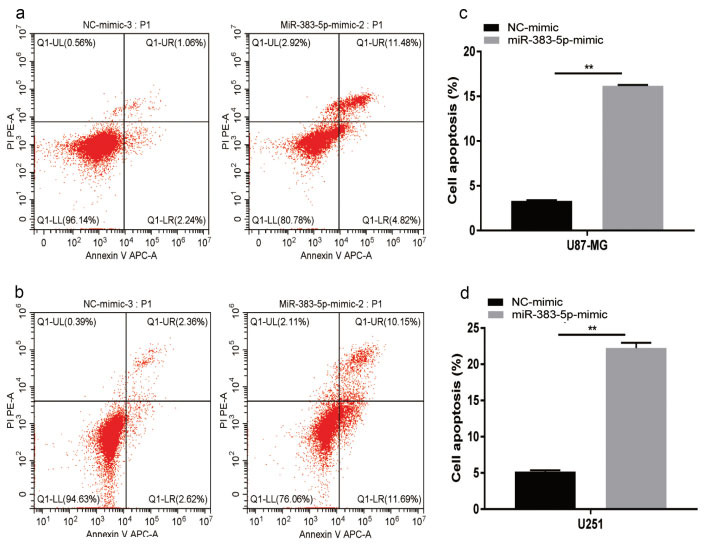
**(a-d)** Flow cytometry analysis of miR-383-5p mimic and NC-mimic added to U87 and U251 cells showed that miR-383-5p mimic led to increased apoptosis in U87 and U251 cells. Each experiment was repeated at least three times. ^∗^*P* < 0.05, ^∗∗^*P* < 0.01.

**Fig. (4):**
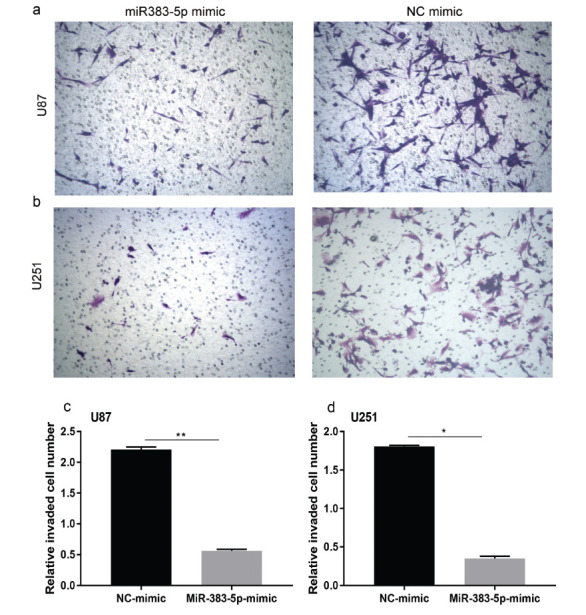
**(a-d)** Transwell migration and invasion assays with U87 and U251 cells transfected with miR-383-5p mimics or NC. Each experiment was repeated at least three times. ^∗^*P* < 0.05, ^∗∗^*P* < 0.01.

We performed colony formation experiments to further confirm the cancer inhibitory effect of miRNA-383-5p on glioma. Tumor proliferation has been found to be reduced in the miRNA-383-5p group compared to the vector group (Figs. **2a - d**). Furthermore, miRNA-383-5p transfection reduced the viability of U87 and U251 cells (Fig. **2e**). Flow cytometry results showed that the miRNA-383-5p mimic significantly enhanced the degree of apoptosis in cells (Figs. **3a - d**). Transwell assay was utilized to evaluate the effect of miRNA-383-5p on the migration and invasion of glioma cells. Overexpression of miRNA-383-5p significantly suppressed the migration and invasion of U251 and U87 cells (Fig. **4a - d**). These findings illustrate the suppressive effect of miRNA-383-5p on glioma cells.

### 3.2 miRNA-383-5p Acts on the Downstream VEGFR2/Akt/mTOR Pathway by Targeting VEGFA

**Fig. (5):**
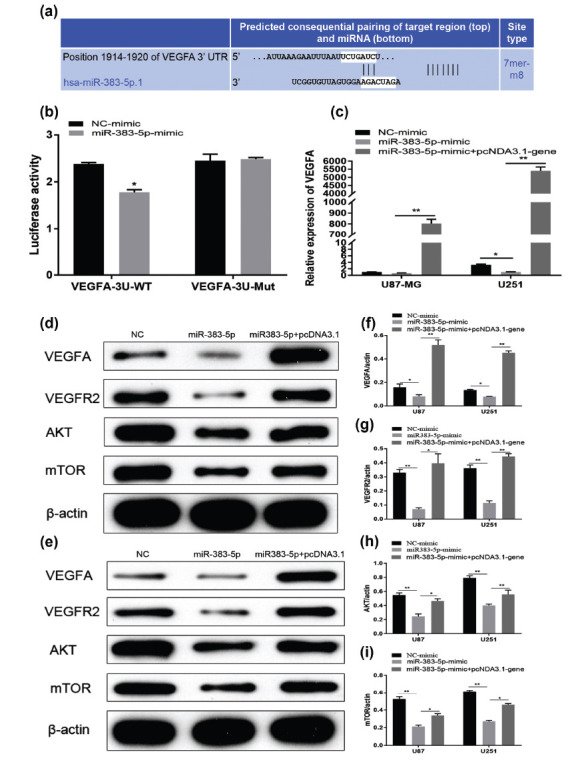
**(a)** The predicted binding sites for miR-383-5p in the 3′-UTR of MYT1L VEGFA and the mutations in the binding sites are demonstrated. (**b**) Relative luciferase activity was measured in HEK293A cells co-transfected with miRNA-383-5p mimic/miRNA-NC and luciferase reporter plasmid. (**c**) qRT-PCR analysis of VEGFA expression in U251 and U87 cells transfected with NC mimic, miR-383-5p, and miR-383-5p+pcDNA3.1-VEGFA. (**d-j**) Western blot assay showing VEGFA, VEGFR2, AKT, and mTOR protein levels in U251 and U87 cells transfected with NC mimic, miR-383-5p, and miR-383-5p+pcDNA3.1-VEGFA. Each experiment was repeated at least three times. ^∗^*P* < 0.05, ^∗∗^*P* < 0.01.

The interesting finding of miRNA-383-5p acting on the downstream VEGFR2/Akt/mTOR pathway by targeting VEGFA has sparked our curiosity to know more about the downstream mechanism of miRNA-383-5p. VEGFA was predicted to be a likely target of miRNA-383-5p based on the putative target sequence in the 3’ UTR (Fig. **5a**). Therefore, we performed a dual-luciferase assay to verify whether VEGFA was a direct target of miRNA-383-5p. Our results demonstrated that miRNA-383-5p inhibited the expression of VEGFA after overexpression of miRNA-383-5p in cells, and miRNA-383-5p did not affect fluorescence activity following VEGFA silencing (Fig. **5b**). The rescue experiment was performed by co-transfection of a miRNA-383-5p mimic with or without a pcDNA3.1-VEGFA plasmid. Overexpression of VEGFA resulted in increased VEGFA mRNA expression in tumor cells compared to controls, and it reversed the VEGFA reduction induced by miRNA-383-5p transfection (Fig. **5c**). Molecular alterations in the PI3K/Akt/mTOR signaling pathway are typical of gliomas. Activation of the PI3K/AKT/mTOR pathway is associated with poor prognosis in glioblastoma (GBM) patients [15]. Secreted VEGFA has been reported to bind to VEGFR2 on endothelial cell membranes to promote angiogenesis [16], and Akt/mTOR is a well-known downstream target of VEGFR2. Therefore, we suspected that the downstream mechanisms of action of miRNA-383-5p and VEGFA may be related to the AKT/mTOR pathway. As shown in (Figs. **5d - f**), we confirmed that miRNA-383-5p transfection alone indeed inhibited the expression of VEGFA/VEGFR2, whereas the expression of VEGFA/VEGFR2, in the miRNA-383-5p + VEGFA pcDNA group, was not different from the tumor control group. Furthermore, we found decreased expression of both AKT and mTOR proteins in U87 and U251 cells in the miRNA-383-5p transfected group compared to the control group, whereas expression of these proteins was increased in VEGFA-overexpressed cells (Figs. **5g - i**). These results suggest that miRNA-383-5p regulates the expression of VEGFR2/Akt/mTOR signaling pathway proteins through VEGFA.

### 3.3 miRNA-383-5p Inhibits Glioma Cell Proliferation and Cell Viability, which can be Rescued by VEGFA Overexpression

**Fig. (6):**
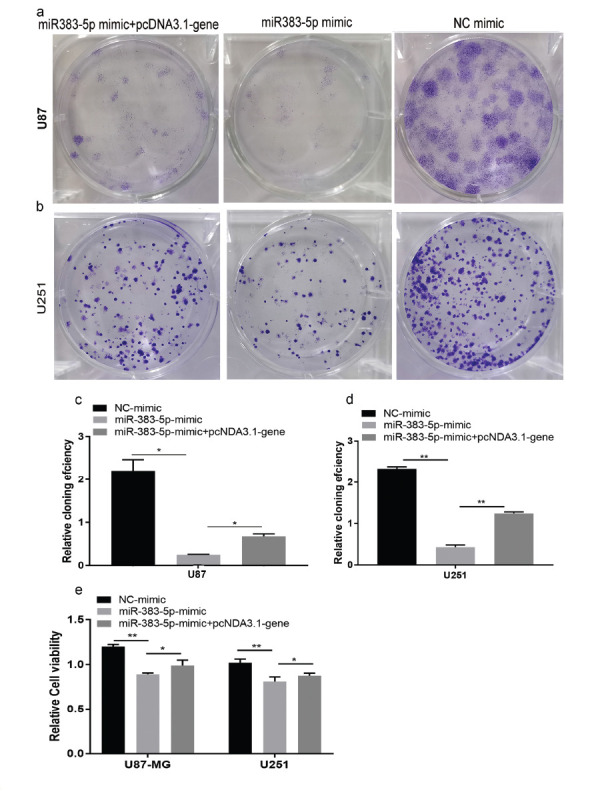
**(a, b)** Comparison of U87 and U251 cell colonies two weeks after transfection of NC mimic, miRNA-383-5p mimic, and miRNA-383-5p+pcDNA3.1-VEGFA. (**c, d**) The reintroduction of VEGFA significantly increased the number of colonies in U87 and U251 cells compared to the miRNA-383-5p mimic alone transfection group. (**e**) CCK8 experiments suggest that overexpression of VEGFA reversed the tumor cell inactivation that was induced by miR-383-5p mimic transfection in U87 and U251 cells. Each experiment was repeated at least three times. ^∗^*P* < 0.05, ^∗∗^*P* < 0.01.

We performed colony formation experiments to further confirm the cancer inhibitory effect of miRNA-383-5p on glioma (Figs. **6a - d**). Tumor proliferation was found to be reduced in the miRNA-383-5p group compared to the vector group. Furthermore, miRNA-383-5p transfection reduced the viability of U87 and U251 cells (Fig. **6e**). These findings illustrate the tumor suppressive effect of miRNA-383-5p in glioma. Overexpression of VEGFA resulted in increased VEGFA mRNA expression in tumor cells compared to controls, and it reversed the VEGFA reduction induced by miRNA-383-5p transfection (Figs. **6a - d**). In addition, the dramatic reduction in cell viability caused by miRNA-383-5p was rescued by overexpression of VEGFA (Fig. **6e**).

### 3.4 miRNA-383-p Regulates Glioma Cell Apoptosis by Targeting VEGFA

**Fig. (7):**
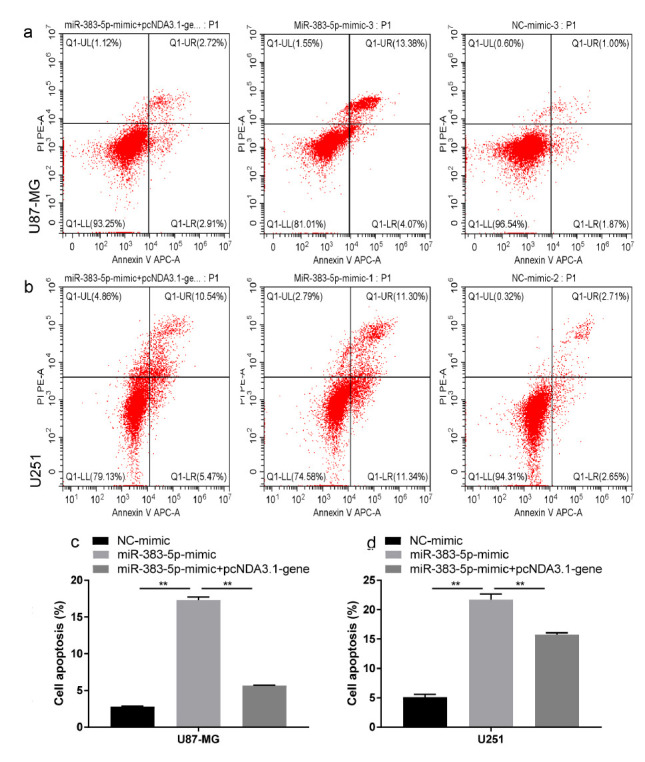
**(a, c)** Flow cytometry results suggest that miR-383-5p mimic transfection in U87 cells led to increased tumor apoptosis, which was partially alleviated by concurrent overexpression of VEGFA. (**b, d**) As in U87 cells, miR-383-5p mimic transfection also resulted in increased tumor cell apoptosis in U251 and U87 cells.

The upregulation of VEGFA is related to the increased apoptosis of glioma cells. We next tested the hypothesis that miRNA-383-p regulates apoptosis through VEGFA. The results of flow cytometry showed that miRNA-383-5p transfection increased the apoptosis of U87 and U251 cells (Figs. **7a - d**). However, this inhibition was greatly reduced by overexpression of VEGFA (Figs. **7a - d**). Collectively, these results suggest that miRNA-383-5p inhibits apoptosis in glioma cells by inhibiting the expression of VEGFA.

### 3.5 miRNA-383-5p Inhibits the Invasion of Glioma Cells, which is Counteracted by Overexpression of VEGFA

**Fig. (8):**
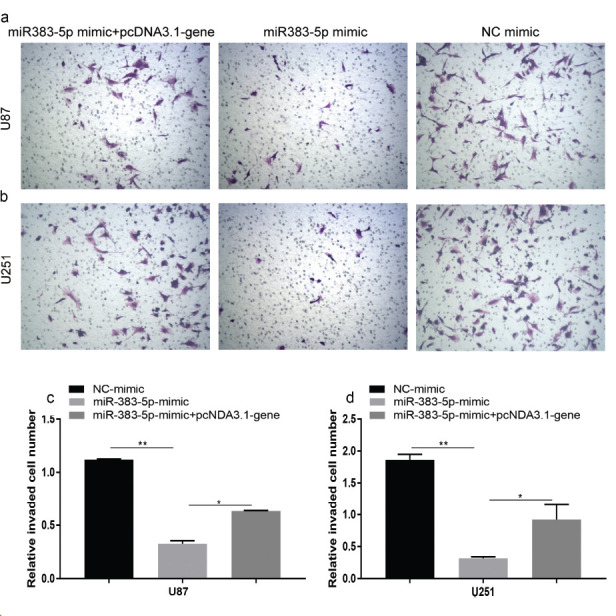
VEGFA expression inhibited the inhibitory effect of miR-383-5p on glioma cells. (**a-d**) Transwell invasion assay was used to assess the number of invaded U87 and U251 cells after transfection of NC mimic, miRNA-383-5p mimic, and miRNA-383-5p+pcDNA3.1-VEGFA.

Since VEGFA has been reported to be associated with increased cellular invasiveness, we hypothesized that miRNA-383-5p regulates tumor invasiveness through VEGFA. Transwell invasion assays showed that miRNA-383-5p transfection reduced tumor cell invasiveness; however, tumor cell invasive functions were restored when miRNA-383-5p transfection and VEGFA overexpression occurred simultaneously (Figs. **8a - d**). This indicates that miRNA-383-5p primarily affects the invasive behavior of tumors by regulating the expression of VEGFA.

## 4 DISCUSSION

In this work, we have reported miRNA-383-5p to act as a glioma suppressor and found it to inhibit aggressive glioma cell biological behavior *via* downstream regulation of VEGFA/mTOR. Overexpression of miRNA-383-5p has been found to inhibit glioma cell proliferation and invasion while promoting apoptosis. We confirmed that the ectopic expression of VEGF1 reversed the inhibitory effect of miRNA-383-5p overexpression on the proliferation, migration, and invasion of glioma cells.

Since the binding of microRNAs to the 3' UTR of the target mRNA is not strictly paired, there are multiple downstream regulatory units. Upregulation of miRNA-383-5p inhibits cell proliferation and tumor growth, and enhances chemosensitivity of ovarian cancer cells by inhibiting TRIM27 expression [17]. LDHA is a target of miRNA-383-5p in gastric cancer [18].

The expression of miRNA-383-5p is downregulated in lung cancer tissues and plays an antiproliferative role by targeting CIP2A [19]. AKR1B10, which is also regulated by miRNA-383-5p, promotes hepatocellular carcinoma (HCC) progression and may be a therapeutic target for precision medicine [20]. In this research, transfection of miRNA-383-5p mimics promoted apoptosis and reduced the proliferation of U87 and U251 cells. The results of our study suggest that the status of miRNA-383-5p is critical for glioma progression. Overexpression of miRNA-383-5p in U87 cells blocks G-S transition and induces apoptosis, suggesting that miRNA-383-5p acts as a tumor suppressor in gliomas. Given that microRNAs act by binding to target mRNAs, we further explored the target of miRNA-383-5p.

We predicted and confirmed that the direct downstream target of miRNA-383-5p is VEGFA in this study. Angiogenesis is essential for supplying the nutrients needed for tumor growth and development and, therefore, is regarded as an important target for cancer therapy. Multiple studies have shown that VEGFA expression is increased in various cancers and contributes to tumor progression at multiple levels, including proliferation, differentiation, and migration [21-23]. Bevacizumab prevents VEGFA from interacting with its receptors, thereby inhibiting downstream signaling pathways. It is the first anti-angiogenic immunotherapy drug approved by the FDA for the treatment of GBM [24]. Although VEGF and VEGFRs have been recognized as therapeutic targets for inhibiting angiogenesis, anti-tumor angiogenesis therapies have shown limited efficacy with survival benefits ranging from only weeks to months. Moreover, other studies have reported tumor progression during antiangiogenic therapy [7]. A growing number of studies tend to combine antivascular therapy with immunotherapy [25-27]. Antiangiogenic agents stimulate the immune system and improve the immunosuppressive environment, and immunotherapy can also have antiangiogenic effects. There is a synergistic relationship between the two treatments [25]. Tumor cells can escape T cell-mediated killing by upregulating the interaction of PD-L1 with the inhibitory receptor PD-1. In breast cancer, Azarbarzin *et al.* found miRNA-383-5p to directly target PD-L1 to inhibit tumor proliferation and migration [28]. Therefore, compared to traditional VEGFA inhibitors, regulating the expression of 383-5p may synergistically inhibit tumor progression from multiple aspects and show better efficacy.

## CONCLUSION

Our study found that miRNA-383-5p is expressed at low levels in gliomas, and is closely related to prognosis and survival of glioma patients. These findings indicate that miRNA-383-5p has clinical diagnostic value. Our findings have also revealed an anti-tumor role of miRNA-383-5p in glioma. The tumorigenesis of glioma is regulated by the miRNA-383-5p direct target VEGFA and the downstream mTOR signaling pathway, which highlights the value of researching VEGFA as a therapeutic target for glioma. miRNA-383-5p is involved in various biological processes related to glioma proliferation, apoptosis, and migration, demonstrating its potential to serve as an effective target for glioma therapy.

## Figures and Tables

**Table 1:** Primers for qRT-PCR.

**Symbol**	**F**	**R**
H-U6	CTCGCTTCGGCAGCACA	AACGCTTCACGAATTTGCGT
H-GAPDH	ACAGCCTCAAGATCATCAGC	GGTCATGAGTCCTTCCACGAT
H-VEGFA	TGCTCTACTTCCCCAAATCACT	ACTCACTTTGCCCCTGTCG
hsa-miR-383-5p	AGATCAGAAGGTGATTGTGGCT	

## Data Availability

The data and supportive information are available within the article.

## LIST OF ABBREVIATIONS

GBM: Glioblastoma
miRNAs: MicroRNAs
VEGFA: Vascular Endothelial Growth Factor A
NC: Negative Control
CGGA: China Glioma Database

## References

[1] Bray F., Ferlay J., Soerjomataram I., Siegel R.L., Torre L.A., Jemal A. (2018). Global cancer statistics 2018: GLOBOCAN estimates of inci-dence and mortality worldwide for 36 cancers in 185 countries.. CA Cancer J. Clin..

[2] Tan A.C., Ashley D.M., López G.Y., Malinzak M., Friedman H.S., Khasraw M. (2020). Management of glioblastoma: State of the art and fu-ture directions.. CA Cancer J. Clin..

[3] Wang L., Liu Y., Yu Z., Gong J., Deng Z., Ren N., Zhong Z., Cai H., Tang Z., Cheng H., Chen S., He Z. (2021). Mir-139-5p inhibits glioma cell proliferation and progression by targeting GABRA1.. J. Transl. Med..

[4] Yu Z., Liu Y., Li Y., Zhang J., Peng J., Gong J., Xia Y., Wang L. (2022). miRNA-338-3p inhibits glioma cell proliferation and progression by targeting MYT1L.. Brain Res. Bull..

[5] Gong J., Tang Z., Yu Z., Deng Z., Liu Y., Ren N., Wang L., He Z. (2022). miR-138-5p inhibits the growth and invasion of glioma cells by regulating WEE1.. Anal. Cell. Pathol..

[6] Zhi Y., Xie X., Wang R., Wang B., Gu W., Ling Y., Dong W., Zhi F., Liu Y. (2015). Serum level of miR-10-5p as a prognostic biomarker for acute myeloid leukemia.. Int. J. Hematol..

[7] Srinivasan S., Patric I.R.P., Somasundaram K. (2011). A ten-microRNA expression signature predicts survival in glioblastoma.. PLoS One.

[8] Romano G., Kwong L.N. (2018). Diagnostic and therapeutic applications of miRNA-based strategies to cancer immunotherapy.. Cancer Metastasis Rev..

[9] Abba M.L., Patil N., Leupold J.H., Moniuszko M., Utikal J., Niklinski J., Allgayer H. (2017). MicroRNAs as novel targets and tools in cancer therapy.. Cancer Lett..

[10] Chen L., Han X. (2015). Anti-PD-1/PD-L1 therapy of human cancer: Past, present, and future.. J. Clin. Invest..

[11] Cortez M.A., Ivan C., Valdecanas D., Wang X., Peltier H.J., Ye Y., Araujo L., Carbone D.P., Shilo K., Giri D.K., Kelnar K., Martin D., Komaki R., Gomez D.R., Krishnan S., Calin G.A., Bader A.G., Welsh J.W. (2015). PDL1 regulation by p53 via miR-34.. J. Natl. Cancer Inst..

[12] Zhou Q., Liu J., Quan J., Liu W., Tan H., Li W. (2018). MicroRNAs as potential biomarkers for the diagnosis of glioma: A systematic review and meta‐analysis.. Cancer Sci..

[13] Gu J., Lu Z., Ji C., Chen Y., Liu Y., Lei Z., Wang L., Zhang H.T., Li X. (2017). Melatonin inhibits proliferation and invasion via repression of miRNA-155 in glioma cells.. Biomed. Pharmacother..

[14] Huang W., Zhong Z., Luo C., Xiao Y., Li L., Zhang X., Yang L., Xiao K., Ning Y., Chen L., Liu Q., Hu X., Zhang J., Ding X., Xiang S. (2019). The miR-26a/AP-2α/Nanog signaling axis mediates stem cell self-renewal and temozolomide resistance in glioma.. Theranostics.

[15] Chakravarti A., Zhai G., Suzuki Y., Sarkesh S., Black P.M., Muzikansky A., Loeffler J.S. (2004). The prognostic significance of phosphati-dylinositol 3-kinase pathway activation in human gliomas.. J. Clin. Oncol..

[16] Potente M., Gerhardt H., Carmeliet P. (2011). Basic and therapeutic aspects of angiogenesis.. Cell.

[17] Jiang J., Xie C., Liu Y., Shi Q., Chen Y. (2019). Up-regulation of miR-383-5p suppresses proliferation and enhances chemosensitivity in ovari-an cancer cells by targeting TRIM27.. Biomed. Pharmacother..

[18] Wei C., Gao J.J. (2019). Downregulated miR-383-5p contributes to the proliferation and migration of gastric cancer cells and is associated with poor prognosis.. PeerJ.

[19] Zhao S., Gao X., Zang S., Li Y., Feng X., Yuan X. (2017). MicroRNA-383-5p acts as a prognostic marker and inhibitor of cell proliferation in lung adenocarcinoma by cancerous inhibitor of protein phosphatase 2A.. Oncol. Lett..

[20] Wang J., Zhou Y., Fei X., Chen X., Chen Y. (2018). Biostatistics mining associated method identifies AKR1B10 enhancing hepatocellular car-cinoma cell growth and degenerated by miR-383-5p.. Sci. Rep..

[21] Wang N., Chen Y., Shi C., Lin Z., Xie H. (2022). CREB3L4 promotes angiogenesis and tumor progression in gastric cancer through regulating VEGFA expression.. Cancer Gene Ther..

[22] Barbagallo D., Caponnetto A., Brex D., Mirabella F., Barbagallo C., Lauretta G., Morrone A., Certo F., Broggi G., Caltabiano R., Barbagallo G., Spina-Purrello V., Ragusa M., Di Pietro C., Hansen T., Purrello M. (2019). CircSMARCA5 regulates VEGFA mRNA splicing and angiogenesis in glioblastoma multiforme through the binding of SRSF1.. Cancers..

[23] Hu F., Sun X., Li G., Wu Q., Chen Y., Yang X., Luo X., Hu J., Wang G. (2018). Inhibition of SIRT2 limits tumour angiogenesis via inactiva-tion of the STAT3/VEGFA signalling pathway.. Cell Death Dis..

[24] Sullivan L.A., Brekken R.A. (2010).

[25] Garber K. (2014). Promising early results for immunotherapy-antiangiogenesis combination.. J. Natl. Cancer Inst..

[26] Hu H., Chen Y., Tan S., Wu S., Huang Y., Fu S., Luo F., He J. (2022). The research progress of antiangiogenic therapy, immune therapy and tumor microenvironment.. Front. Immunol..

[27] Lopes-Coelho F., Martins F., Pereira S.A., Serpa J. (2021). Anti-angiogenic therapy: Current challenges and future perspectives.. Int. J. Mol. Sci..

[28] Azarbarzin S., Hosseinpour-Feizi M.A., Banan Khojasteh S.M., Baradaran B., Safaralizadeh R. (2021). MicroRNA -383-5p restrains the prolif-eration and migration of breast cancer cells and promotes apoptosis via inhibition of PD-L1.. Life Sci..

